# Acute kidney injury through a metabolic lens: pathological reprogramming mechanisms and clinical translation potential

**DOI:** 10.3389/fphys.2025.1602865

**Published:** 2025-06-06

**Authors:** Jingli Gao, Liuyifei Huang, Yuzhan Zhang, Lei Wei, Zhixiang Yu, Yan Xing, Jinguo Yuan, Xiaoxuan Ning, Shiren Sun

**Affiliations:** ^1^ Department of Nephrology, Xijing Hospital, Fourth Military Medical University, Xi’an, China; ^2^ Department of Geriatric, Xijing Hospital, Air Force Medical University, Xi’an, China

**Keywords:** acute kidney injury, fatty acid oxidation, glycolysis, BCAAs, ketolysis, glutaminolysis

## Abstract

Acute kidney injury (AKI) represents a clinical syndrome with a bleak short-term prognosis, posing a high risk for the development of chronic kidney diseases and end-stage kidney disease. The underlying mechanisms of AKI are still not fully understood, and effective intervention strategies remain elusive. Enormous energy is required to meet the functional activity in hypermetabolic tubular epithelial cells (TECs), the most vulnerable cell types during AKI. Recent evidence has shed light on the reprogramming of metabolic pathways and the shift in energy substrates under pathological conditions. The reprogrammed metabolic pathway initially serves to compensate for energy shortages and supply substrates for cell repair during the early stages of AKI. However, sustained metabolic dysregulation tend to become detrimental for tubular repair and regeneration. Intriguingly, dynamic alterations in specific metabolites extend beyond their conventional roles as metabolic byproducts, actively participating in pathophysiology through multifaceted regulatory mechanisms during AKI. As yet, clinical therapy for AKI has not yet incorporated the intervention of metabolic disorders, highlighting a vast potential for extensive application. This review aims to summarize recent studies on the role of metabolic pathway reprogramming and metabolites in AKI, while discussing promising therapeutic strategies targeting metabolic reprogramming.

## 1 Introduction

Acute kidney injury (AKI) is characterized by an abrupt deterioration of kidney function, manifested by elevated serum creatinine levels and decreased urinary output within a 7-day period. The etiologies of AKI are categorized into pre-renal, renal, and post-renal factors, primarily involving ischemia/reperfusion injury (IRI), toxins, and urinary obstruction (Kellum et al., 2021). Patients with AKI face an 8.8-fold higher risk of developing chronic kidney disease (CKD), a 3.1-fold higher risk of end stage renal disease (ESRD), and a 2.0-fold higher risk of mortality compared to non-AKI populations (Coca et al., 2012). Among the most abundant intrinsic renal cells, tubular epithelial cells (TECs) are extremely sensitive and susceptible to AKI. The fate of TECs and kidney tissue is influenced by various pathological processes, including but not limited to inflammation (Matsushita et al., 2020), oxidative stress (Aranda-Rivera et al., 2021), cell cycle arrest (De Chiara et al., 2021), epigenetic regulation (Guo et al., 2019), and gut microbiota dysbiosis (Saranya and Viswanathan, 2023).

In recent decades, there has been a growing focus on the role and function of cellular metabolism of organs in disease processes. Maintaining metabolic homeostasis is fundamental requirement for cell growth, proliferation, and the specialized physiological functions (O'Brien et al., 2020). TECs have high metabolic activity and primarily rely on fatty acid oxidation (FAO) rather than glucose oxidation for their energy supply under physiological conditions. Complementary energy substrates including amino acids (AAs), ketone bodies, pyruvate, and lactate further contribute as fuel for aerobic respiration to generate adenosine triphosphate (ATP) in tubular cells (Scholz et al., 2021). Pathological stress induces profound metabolic alterationscharacterized by mitochondrial dysfunction and an imbalance of 5′-adenosine monophosphate (AMP)-activated protein kinase (AMPK) and mammalian target of rapamycin (mTOR) in TECs. This metabolic reprogramming recapitulates the embryonic development and differentiation patterns observed in nephron progenitor cells (NPCs) which initially serving as an adaptive response. Paradoxically, sustained metabolism reprogramming contributes to maladaptive repair of TECs and deterioration in kidney function (Zhu et al., 2022; Wang G. et al., 2022).

Apart from metabolic disorders, endogenous intermediate metabolites play a role not only as products of the metabolic process but also as influencers of the outcome of multiple tissues during nutrient stress. Endogenous intermediate metabolites exert multifaceted regulatory effects through two principal mechanisms, (1) transducing signaling pathways by binding with receptors, (2) covalently modification of amino acid residues via post-translational modifications (PTMs) which may affect protein localization, conformational properties, and intermolecular interaction networks by competing with other binding partners (Figlia et al., 2020; Wang and Lei, 2018).

Currently, there are few effective or potent therapies available for halting the progression of AKI. Given that the dysregulation of metabolic homeostasis considerably disrupts cells and tissues, exploring key metabolic pathways and metabolites may yield novel biomarkers and therapeutic strategies for AKI. In this review, we aim to elucidate how reprogrammed metabolic pathways, along with the differential metabolites, dedicate TECs fate and renal outcome in AKI, with particular emphasis on their translational potential for AKI management (Table 1).

**TABLE 1:** Roles of metabolic pathways and metabolites in signal transduction, protein modifications, and the effects on AKI.

Metabolic pathways	Metabolites	Variation tendency	Roles and mechanisms in AKI
FAO		Suppressed	FAO enzyme CPT1A overexpression prevented kidney fibrosis, mitochondrial morphology impairment and M1 macrophage infiltration in folic acid-induced AKI (Miguel et al., 2021)
	butyrate	Decreased in serum in LPS-induced AKI (Gao et al., 2021)	Activates apoptosis, suppresses ROS production in TECs, suppresses activation of inflammatory cells in IRI-induced AKI (Andrade-Oliveira et al., 2015)
	crotonate	NA	Protects kidney by increasing histone Kcro/PGC-1α/SIRT3 in folic acid- or cisplatin-induced AKI (Ruiz-Andres et al., 2016)
	palmitate	Increased in kidney and serum in IRI, UUO-, cisplatin- induced AKI (Li H. et al., 2022; Huang et al., 2018; Qu et al., 2020)	Increases OCR and ECAR in RPTEC (Li H. et al., 2022) Enhances fibrosis by β-catenin palmitoylation in UUO- and IR-induced kidney injury (Gu et al., 2023)
TCA cycle		Suppressed	Inhibiting enzyme PDK4 mitigates oxidative stress, elevates mitochondrial membrane potential and ATP production, reduces mitochondrial fragmentation in IR-induced kidney injury (Oh et al., 2023) Inhibiting enzyme PDK reduces kidney fibrosis, tubular apoptosis, and macrophage infiltration in UUO-induced injury (Wei et al., 2019)
	succinate	Increased in kidney in IRI-induced AKI	Produces ROS and worsening of kidney injury in IR-induced AKI (Oh et al., 2023) Promotes apoptosis by activating SUCNR1/ERK in HK-2 cells, induces apparent renal injury in succinate-treated mice (Pu et al., 2023) Activates the RAS system by activating SUCNR1/NO/PGE2 in JGA (Deen and Robben, 2011) Polarizes macrophages by activating the SUCNR1/PI3κ/HIF-1α axis (Wu et al., 2020) Blocks mitochondrial FAO and increases peroxisome FAO by increasing succinylation in cisplatin-induced AKI (Chiba et al., 2019)
Ketolysis		Suppressed	NA
	β-OHB	Increased in urine in IRI-induced AKI (Jouret et al., 2016)	Induces production of renoprotective PGE2 (Tran et al., 2016) Antagonizes oxidative stress by FOXO3/HDAC/Histone acetylation (Shimazu et al., 2013) Anti-pyroptotic effects by increasing expression of FOXO3 in IRI-induced AKI (Tajima et al., 2019) Suppresses NLRP3 inflammasome in cisplatin-induced AKI (Luo et al., 2022) Reduces tubular apoptosis and inflammatory responses by reducing p-NF-κB p65 in LPS-induced AKI (Kim et al., 2023) Mitigates glomerulosclerosis by upregulating H3K9 Kbhb/MMP-2 in DKD rats (Luo et al., 2020) Reduces cell growth arrest and apoptosis by p53 Kbhb in HEK-293 cells (Liu K. et al., 2019)
BCAAs catablolism		Suppressed	Enhancing BCAA catabolism by BT2 increases OCR in HK-2 cells (Piret et al., 2021) Inhibiting BCAA catabolic enzyme BCKDHB reduces mitochondrial ATP synthesis in HK-2 cells (Piret et al., 2021) Inhibiting BCAA catabolic enzyme BCAT1 improves kidney function by suppressing the infiltration of macrophage in crescentic glomerulonephritis rats (Papathanassiu et al., 2017)
	BCAAs	Increased in kidney in UUO- and LPS-induced AKI (Li H. et al., 2022; Xu et al., 2023), increased in urine in cisplatin-induced AKI (Pariyani et al., 2017)	Enhances cells proliferation by activating mTOR/MAPK/ERK pathways in polycystic kidney disease (Yamamoto et al., 2017) Reduces GFR and renal plasma flow, increase plasma free FAs and kidney smooth muscle actin (α-SMA), collagen level in 5/6 nephrectomy rats (Pillai et al., 2019) Reduces kidney glomeruli number in a doxorubicin toxicity model (Duarte et al., 2023) Downregulated serum creatinine at day 28 in UUO-induced AKI (Jouret et al., 2016)
Tryptophan metabolism		Suppressed (NAD biosynthesis)	Affects metabolic disorder by reducing NAD production in IR-induced AKI (Poyan Mehr et al., 2018)
	Kyn, IS, IAA	Increased in urine and plasma in AKI patients (Aregger et al., 2018; Zhao, 2013)	Increases oxidative stress and inflammation by activating AhR (Hui et al., 2023)
Glycolysis		Enhanced	Inhibiting glycolytic enzyme HK promotes autophagy, suppresses apoptosis and improves kidney function by enhancing SIRT3/AMPK pathway in LPS-induced AKI (Tan et al., 2021) Inhibiting glycolytic enzyme PKM2 reduces kidney fibrosis, tubular apoptosis, and macrophage infiltration in UUO-induced injury (Wei et al., 2019)
	Lactate	Increased in CPB/DHCA-, IR-, CLP-induced AKI (Davidson et al., 2022; Lan et al., 2016; Tan et al., 2021)	Inhibits autophagy and enhance apoptosis by inactivating SIRT3/p-AMPK pathway in LPS-treated HK-2 cells (Tan et al., 2021) Suppresses the immuno-response by PD-L1 in sepsis-induced AKI in mice (Xu et al., 2021) Increases mitochondrial fission and ATP depletion by Fis1 K20la in SAKI mice (An et al., 2023) Activates NLRP3 inflammasome and promotes AKI-CKD transition by enhancing renal protein lactylation (Yu et al., 2023) Induces M2-like characteristics and a switch to repairing stage by increasing histone lactylation in macrophages (Zhang et al., 2019) Suppresses glycolysis and polarizes macrophage to reparative phenotype by lactylation of PKM2 in macrophages (Wang J. et al., 2022)
PPP		Enhanced	Inhibiting PPP enzyme G-6-PDH increases proteinuria, and oxidative stress by decreasing NADPH and GSH levels in mice (Xu et al., 2010)
	NADPH	Increased	Increased fatty acid and cholesterol synthesis may lead to lipotoxicity by increased reductive agent NADPH (Scantlebery et al., 2021) Protects kidney function and mitigates the damage bringing by ROS by increasing reductive agent NADPH in IR-induced injury (Zhou et al., 2019)
glutaminolysis		Enhanced	Upregulates GLS activity during ischemia and declines during reperfusion. Applying a GLS antagonist mitigated both IRI- and cisplatin-induced kidney function deterioration by inhibiting the activation and proliferation of T cells (Lee et al., 2023)
	Gln	Decreased in renal cortex and plasma in IRI- and in LPS-induced AKI (Xu et al., 2023; Wei et al., 2014), but increased in CLP- and LPS-induced AKI (Gao et al., 2021; Izquierdo-Garcia et al., 2019)	Decreases kidney damage biomarkers in cardiac surgery patients at high risk of AKI (Weiss et al., 2023) Attenuates tubular cell mitochondrial intrinsic apoptosis by modulating the Tgm2/HSP70/Ask1/JNK pathway in IRI-induced AKI (Thomas et al., 2022) Reduces oxidative stress in gentamicin-induced AKI in rats (Zhan et al., 2022) Protects kidney function by regulating the miRNA/PI3κ/Akt signaling pathway in IR-induced AKI (Li S.et al., 2022) Reduces OCT2 expression and the absorption of cisplatin in HK-2 cells (Kim et al., 2015)
Polyamine catabolism		Enhanced	Inhibition of catabolic enzyme AOC1 reduces cortical tubules with casts and downregulates injury markers in IR-induced AKI (Sieckmann et al., 2023) Inhibition of catabolic enzyme SAT reduces inflammatory reactions, reduces neutrophil infiltration, TNF-α, MCP-1, and IL-6 expression in IR-induced AKI (Zahedi et al., 2014) Inhibition of catabolic enzyme SAT or SMOX reduces endoplasmic reticulum stress response in cisplatin-induced AKI (Zahedi et al., 2017) Overexpression of catabolic enzyme SAT increases oxidative stress in IR-induced AKI (Wang et al., 2004)
	spermidine	Decreased in tetracycline treated HEK-293 cells (Wang et al., 2004), increased in kidney in LPS-induced AKI (Xu et al., 2023), increased in urine in cardiac surgery-AKI (Martin-Lorenzo et al., 2021)	Protects against kidney injury and suppresses NLRP3 inflammasome in macrophages by enhancing mitochondrial respiration capacity in LPS-induced AKI (Li X.et al., 2022)

Abbreviations: AhR, activate aromatic hydrocarbon receptor; AKI, acute kidney injury; Akt, serine/threonine-specific protein kinase; AOC, amine oxidase copper-containing 1; AMPK, AMP-activated protein kinase; α-SMA, smooth muscle actin; Ask1, apoptosis signal-regulating kinase 1; BCAAs, branched-chain amino acids; BCAT1, BCAAs, aminotransferase; BCKDHB, branched chain keto acid dehydrogenase E1 subunit beta; β-OHB, β-hydroxybutyrate; CKD, chronic kidney disease; CPB/DHCA, cardiopulmonary bypass with deep hypothermic circulatory arrest; CLP, cecal ligation and puncture; DKD, diabetic kidney disease; ECAR, extracellular acidification rate; ERK, extracellular signal-regulated kinase; FAs, fatty acids; FAO, fatty acid oxidation; Fis1, fission1; FOXO3, forkhead box O3; G-6-PDH, glucose‐6‐phosphate 1‐dehydrogenase; GSH, gluthionine; HDAC, histone deacetylases; HIF, hypoxia induced factor1α; HSP70, heat shock protein 70; IAA, indole acetic acid; IR, ischemia reperfusion; IRI, ischemia reperfusion injury; IL-6, interleukin-6; IS, indole sulfate; JGA, juxtaglomerular apparatus; JNK, c-Jun N-terminal kinase; Kbhb, lysine hydroxybutyrylation; Kcro, lysine crotonylation; K20la, lysine20 lactylation; Kyn, kynurenine; LPS, lipopolysaccharide; MAPK, mitogen-activated protein kinase; MCP-1, monocyte chemoattractant protein-1; MMP-2, metalloproteinase-2; mTOR, mammalian target of rapamycin; NA, not available; NAD, nicotinamide adenine dinucleotide; NADPH, nicotinamide adenine dinucleotide phosphate; NLRP3, nucleotide-binding oligomerization domain leucine-rich repeat and pyrin domain-containing 3; NO, nitric oxide; OCR, oxygen consumption rate; OCT2, organic cation transporter2; PD-L1, Programmed cell death 1 ligand 1; PDK, pyruvate dehydrogenase kinase; PGC-1α, peroxisome proliferator-activated receptor-gamma coactivator-1α; PGE2, prostaglandin2; PI3κ, phosphatidylinositol-3-kinase; PKM2, pyruvate kinase M2; PPP, pentose phosphate pathway; ROS, reactive oxygen species; SAKI, sepsis acute kidney injury; SAT, spermidine/spermine N1-acetyltransferase; SIRT3, sirtuin 3; SMOX, spermine oxidase; SUCNR1, succinate receptor 1; TECs, tubular epithelial cells; Tgm2, transglutaminase 2; TNF-α, tumor necrosis factor-α; UUO, unilateral ureteral obstruction.

## 2 Downregulated metabolic pathways

### 2.1 Fatty acid oxidation

**FIGURE 1:**
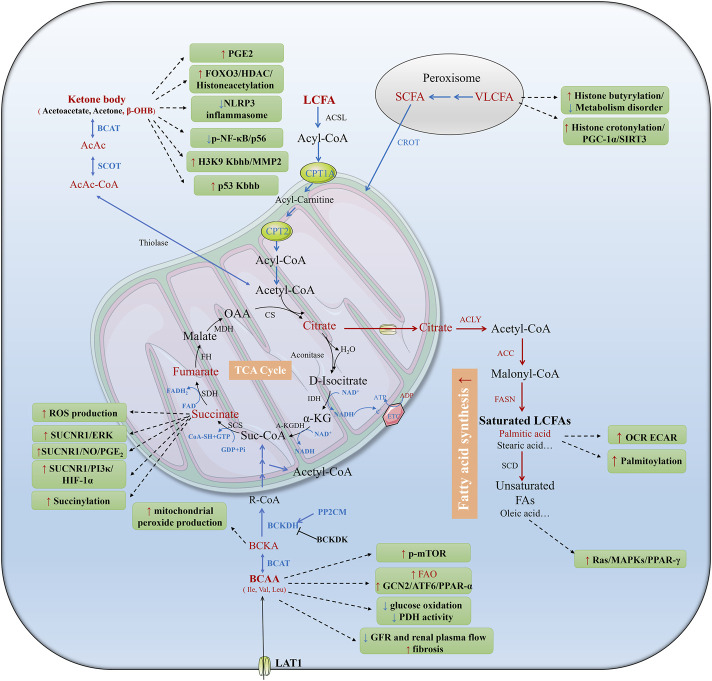
Metabolic reprogramming of tubular epithelial cells (TECs) during AKI. AKI can be induced by hypoxia, toxins, or urinary obstruction. AKI results in mitochondrial dysfunction and low oxygen availability which impedes the electron transport chain and aerobic respiration. The shift from FAO to glycolysis is widely studied in AKI. In addition, ketolysis, BCAA catabolism, glutaminolysis, polyamine metabolism and their metabolites play different roles in AKI. Red and blue fonts and arrows mean up- and downregulated pathways and metabolites in AKI, black fonts and arrows mean unidentified pathways and metabolites in previous studies. BCAAs, branched-chain amino acids; Suc-CoA, succinyl coenzyme; acetyl-CoA, acetyl coenzyme; acyl-CoA, acyl coenzyme; TCA, tricarboxylic acid; Gln, glutamine; Glu, glutamate; α-KG, α-ketoglutarate; NAD, nicotinamide adenine dinucleotide; FAD, flavin adenine dinucleotide; NADPH, nicotinamide adenine dinucleotide phosphate; G-6-P, glucose-6-phosphate; PPP, pentose phosphate pathway; FA, fatty acid; FAO, fatty acid oxidation; FAS, fatty acid synthesis; CPT1, carnitine palmitoyl-transferase 1; CPT2, carnitine palmitoyl-transferase 2; Try, tryptophan; QUIN, quinolinic acid; ATP, adenosine triphosphate; ADP, adenosine diphosphate; ETC, electron transport chain; e^−^, electron; GABA, γ-aminobutyric acid; Put, putrescine; Spd, spermidine; Spm, spermine. GSSG, oxidized glutathione; GSH, reduced glutathione; H_2_O_2_, hydrogen peroxide.

Fatty acids (FAs) are transported by the CD36 membrane glycoprotein, fatty acid binding protein (FABP), and fatty acid transport protein (FATP) in TECs. In the mitochondrial membrane, long-chain fatty acids (LCFAs, C_18_ ≤ C_22_), medium-chain fatty acids (MCFAs, C_6_≤C_12_), and short-chain fatty acids (SCFAs, ≤C_6_) are catalyzed by long/medium/short-chain acyl-CoA synthetase (ACSL, ACSM, ACSS) to produce acyl coenzyme A (acyl-CoA) (Figure 1). Carnitine palmitoyl-transferase 1A (CPT1A) acts as a crucial, speed-limiting enzyme on the inner membrane of mitochondria. It catalyzes the transformation of acyl-CoA from coenzyme A to L-carnitine, generating acyl-carnitine to facilitate the transfer of fatty acids from cytosol to the mitochondria. Then palmitoyl-transferase 2 (CPT2) reversely releases acyl-carnitine, reverting it to acyl-CoA and carnitine in mitochondrial matrix. Subsequently, acyl-CoA undergoes stepwise oxidation to acetyl coenzyme A (Ac-CoA), which then enters the tricarboxylic acid (TCA) cycle. This cycle produces nicotinamide adenine dinucleotide (NADH) and flavin adenine dinucleotide (FADH2), both participating in oxidative phosphorylation to produce ATP and H_2_O. Additionally, FAO can also take place in peroxisomes, which mainly catalyze very long chain fatty acids (VLCFAs, ≥C_22_) into SCFAs. These degraded SCFAs can be transferred to mitochondria or cytosol to be utilized or secreted.

#### 2.1.1 Suppressed FAO during AKI

During AKI, mitochondrial dysfunction can disrupt the electron transport chain (ETC). Ischemia-induced low oxygen availability hampers electron transport in the ETC, ultimately inhibiting substrate oxidation in the TCA cycle. Consequently, upstream FAO is also halted. Applying single-cell combinatorial indexing RNA sequencing in uIRI (unilateral ischemia reperfusion injury) and UUO (unilateral ureteral obstruction) mice, Li et al. found a negative correlation between the proportion of failed-repaired proximal tubular cells (FR-PTC) and FAO activity (Li H. et al., 2022). Lipid droplet accumulation significantly increased in uIRI at 6 h on Day (D)2 and decreased nearly to baseline at D7 and D14. By contrast, lipid droplets gradually accumulated gradually over time in UUO mice (Li H. et al., 2022). Kidney transplant recipients who underwent severe IRI were found to have enrichment of LCFAs in their urine (Rinaldi et al., 2022).

Researchers observed that kidney CD36 scavenger receptor significantly increased in patients with AKI and in mice with cisplatin-induced AKI (Ma et al., 2024). Tubule-specific CD36 overexpression exacerbated proteinuria and fibrosis in mice with folic acid-induced AKI (Jung et al., 2018), indicating that increased CD36 was associated with the progression of AKI. A study revealed that kidney CPT1A levels in patients with CKD, and kidney CPT2 levels in patients post-kidney transplantation were significantly correlated with eGFR (Miguel et al., 2021). In addition, serum levels of short- and middle-chain acylcarnitines were elevated in patients with CKD group compared to healthy controls (Miguel et al., 2021), indicating a reduction of fatty acid transportation into the mitochondria. Treatment with the CPT1 inhibitor, etomoxir, suppressed ATP production, elevated apoptosis, and led to dedifferentiation in TECs (Kang et al., 2015). In a study by Miguel et al., tubule-specific overexpression of CPT1A in mice prevented kidney fibrosis, mitochondrial morphology impairment, and M1 macrophage infiltration induced by folic acid nephropathy (Miguel et al., 2021). Furthermore, the speed-limiting enzyme for FAO, acyl-coenzyme A oxidase 1 (ACOX), was found to be decreased after IR-induced injury in TECs, showing a negative correlation with kidney function (Chen et al., 2023). Carnitine O-Octanoyltransferase (CROT), responsible for transferring fatty acids from peroxisomes to mitochondria for FAO, and alpha-methylacyl-CoA racemase (AMACR), involved in peroxisome β-oxidation, were also decreased in IR-induced AKI (Chen et al., 2023). These studies collectively suggest the suppression of both mitochondrial and peroxisomal FAO.

#### 2.1.2 Metabolites of FAO

The intermediate carboxylic acids generate several acyl-CoAs, and these acyl-CoAs could emerge as “donors” transferred to AA residuals, such as lysine, by acyltransferase or removed by deacylase. Acylated proteins resulting from these processes can have an impact on various signaling functions. While enzymes like lysine acetyltransferase (KAT) and histone deacetylase (HDAC), responsible for adding and removing acetyl-CoA, have been extensively studied in the past, it is essential to recognize that the abundance of metabolites can also affect post-translational modification either enzymatically or non-enzymatically (Simithy et al., 2017;Sabari et al., 2017).

As a LCFA, palmitate accumulated in both kidney and serum during AKI induced by IRI, UUO, and cisplatin (Li H. et al., 2022; Huang et al., 2018; Qu et al., 2020). Palmitate treatment has been shown to increase both the oxygen consumption rate and extracellular acidification rate simultaneously (Li H. et al., 2022), indicating an enhancement of FAO and glycolysis at the same time in human renal proximal TECs. However, Gu et al. observed a decrease in palmitoyltransferase which significantly downregulated palmitoylation of β-catenin and delayed degradation of β-catenin, ultimately contributing to enhanced fibrosis in both UUO- and IR-induced AKI in mice, as well as in TGF-β1 stimulated TECs (Gu et al., 2023). Oleic acid, an unsaturated LCFA, could have a protective effect by mitigating inflammation and oxidative stress through the Ras/MAPKs/PPAR-γ signaling pathway in lipopolysaccharide (LPS)-induced AKI (Zhang B. et al., 2022).

SCFAs, including butyrate, crotonate, malonate, acetate, etc., are composed of 1-6 carbon atoms. SCFAs are mainly derived from the intestinal microbiota but can also originate from the β-oxidation of fatty acids and fatty acid synthesis in cytosol. They can bind with the G protein-coupled receptors and regulate pathophysiological processes (Pluznick, 2014). The levels of SCFAs, especially butyrate, were found to be significantly lower in patients with CKD compared to healthy individuals (Wang et al., 2019). Additionally, serum butyrate levels was significantly downregulated in LPS-induced AKI models. (Gao et al., 2021). Butyrate treatment has demonstrated protective effects on the kidneys during IR-induced injury by activating apoptosis, suppressing reactive oxygen species production in TECs, and suppressing the activation of inflammatory cells (Andrade-Oliveira et al., 2015). Systemic administration of sodium butyrate increased histone butyrylation, ameliorated lipid and glucose metabolic disorders, and attenuated renal inflammation and fibrosis, while the protective effect could be eliminated by histone modification enzyme p300 inhibitor A485 in diabetic kidney disease (DKD)mice (Zhou et al., 2022).However, the roles of butyrate and butyrylation need to be further elucidated in AKI.

As a byproduct of fatty acid β-oxidation, crotonate and histone crotonylation were found to coincide periodically with the expression of fatty acid β-oxidation genes in yeast (Gowans et al., 2019), and their levels could be regulated by FAO enzymes (Zhang Y. et al., 2022). Conversely, Ruiz-Andres et al. demonstrated an increase in histone crotonylation in folic acid- and cisplatin-induced AKI (Ruiz-Andres et al., 2016). The administration of crotonate was shown to upregulate histone crotonylation, prevent the reduction of peroxisome proliferator-activated receptor-gamma coactivator-1α (PGC-1α), and maintain sirtuin 3 (SIRT3) expression, ultimately offering protection against AKI (Ruiz-Andres et al., 2016).

#### 2.1.3 Metabolites in TCA cycle

Low oxygen levels impede oxidative phosphorylation, resulting in the accumulation of intermediate metabolites in the TCA cycle. Renal metabolites in the TCA cycle, including succinate and cis-aconitate, are increased in UUO-induced AKI (Zhao et al., 2016). Additionally, serum levels of citrate, succinate, and fumarate, along with kidney succinate, are increased in cisplatin-induced AKI (Qu et al., 2020).

Succinate that accumulated during hypoxia underwent oxidation during the reperfusion stage, leading to a rapid induction of reactive oxygen species in multiple tissues (Chouchani et al., 2014). Pharmacological inhibition of succinate accumulation has been shown to protect the heart, brain (Chouchani et al., 2014), and kidneys (Oh et al., 2023) from IRI. Succinate can also be secreted into the interstitium, activating succinate receptor 1 in various cell types and triggering several signaling pathways, including upregulating extracellular signal-regulated kinase and promoting apoptosis in HK-2 cells (Pu et al., 2023). This induces the secretion of nitric oxide and prostaglandin2 (PGE2), thereby activating the renin-angiotensin system in the juxtaglomerular apparatus (Deen and Robben, 2011), and affecting macrophage polarization through the phosphatidylinositol-3 kinase/hypoxia-induced factor-1α axis (Wu et al., 2020). Succinate and α-ketoglutaric acid can also serve as “donors” for lysine succinylation, which has been found to increase during IR-induced AKI. Sirtuin5 serves as a succinylation deacylase, and sirtuin5 knockout mice exhibited hypersuccinylation, protecting mice from cisplatin-induced AKI (Chiba et al., 2019). This protection was attributed to the blockage of mitochondrial FAO and the augmentation of peroxisome FAO, which reduced oxidative stress and compensated for the energy shortage in TECs.

Ac-CoA, acetate, and α-ketoglutaric acid are more than intermediate metabolites during aerobic respiration, also participating in covalent donors to regulate protein lysine acetylation (Sivanand et al., 2018). Acetylation is a transient and reversible protein modification, highly correlated with the concentration of these “donors” (Trefely et al., 2020). Kidney tissue acetate concentration is reduced in rats with UUO-induced AKI (Zhao et al., 2016). One study showed a decrease in histone acetylation in kidneys exposed to IRI and in HK-2 cells subjected to hypoxia/reoxygenation (Tajima et al., 2019). Transforming growth factor β1 stimulation induced a shift from aerobic respiratory to glycolysis, leading to a reduction in Ac-CoA concentration and histone 3 acetylation, thereby activating renal myofibroblasts and causing renal fibrosis (Smith et al., 2019). On the other hand, Hewitson et al. found that global acetylation of histone H3 at lysine 9 (H3K9) increased in TECs after UUO compared with a control group (Hewitson et al., 2017). Beyond histones, acetylation also took place in nonhistone proteins such as p53, with increased acetylation observed in sepsis-induced AKI mice. This acetylation of p53 suppressed autophagy and exacerbated tubular injury (Sun et al., 2021). These findings underscore that the availability of Ac-CoA, along with the activity and expression of histone acetyltransferases and histone deacetylases, are predominant factors influencing protein acetylation.

### 2.2 Ketone body oxidation

Ketone bodies comprise acetoacetate, acetone, and β-hydroxybutyrate (β-OHB), generated through the degradation of fatty acids in the liver and subsequently transported to peripheral tissues for utilization as a fuel source. In peripheral tissues, β-OHB dehydrogenase 1 (BDH1) oxidizes β-OHB to acetoacetate. Acetoacetate is then activated to acetoacetyl-CoA by succinyl-CoA:3 oxoacid-CoA transferase (SCOT), and further catalyzed by acetyl-CoA acetyltransferase (ACAT) to produce two molecules of Ac-CoA.

Some evidence suggests that ketolysis might be suppressed in AKI. Proteomic analysis revealed a significant decrease in the levels of ketone body oxidative enzymes, including BDH1, SCOT, and ACAT1 in the kidneys of mice with LPS-induced AKI (Xu et al., 2023). Single cell RNA sequencing showed an initial decrease followed by a gradual recovery in the gene expression of *Bdh1*, *Acat1*, and *Oxct1* (SCOT) in the proximal tubule of uIRI mice (Kirita et al., 2020). In contrast, these genes exhibited a continuous decrease from the outset in UUO mice (Li H. et al., 2022) (single cell sequencing database: http://humphreyslab.com/SingleCell/). Consistent with these findings, urine β-OHB was significantly increased in IRI-induced AKI mice (Jouret et al., 2016), and urine acetoacetate levels were elevated in cisplatin-induced AKI rats (Pariyani et al., 2017).

In addition to its involvement in ketolysis, the metabolite β-OHB also interacted with AKI. Administration of β-OHB protects kidneys via various mechanisms. These include inducing renoprotective PGE_2_ production (Tran et al., 2016), inhibiting histone deacetylase and increasing global histone acetylation, further increasing forkhead box O3 (FOXO3) and antagonizing oxidative stress (Shimazu et al., 2013). β-OHB suppresses the mTOR signal pathway in DKD mice (Tomita et al., 2020), and exhibits anti-pyroptotic effects by increasing expression of FOXO3 in IR-induced AKI (Tajima et al., 2019). Moreover, β-OHB reduced the inflammatory response, oxidative stress, and tubular injury by decreasing Phospho-nuclear factor kappa-light-chain-enhancer of activated B cells subunit 65 (p-NF-κB p65) expression in LPS-induced AKI, and by suppressing the nucleotide-binding oligomerization domain leucine-rich repeat and pyrin domain-containing 3 (NLRP3) inflammasome in Cisplatin-induced AKI (Kim et al., 2023; Luo et al., 2022; Kim et al., 2024). β-OHB also plays signaling roles by inducing lysine hydroxybutyrylation (Kbhb) (Xie et al., 2016) in both histone and non-histone proteins. Administration of β-OHB increased H3K9 Kbhb, upregulated metalloproteinase-2 (MMP-2) expression and downregulated collagen Ⅳ levels, mitigating glomerulosclerosis in DKD rats (Luo et al., 2020). Additionally, β-OHB increased p53 Kbhb, inactivating p53 and resulting in the reduction of cell growth arrest and apoptosis in cultured cells (Liu K. et al., 2019). Although the protective effects of β-OHB in AKI are emerging, the characteristics and roles of ketolysis and Kbhb remain unclear in AKI and require further exploration.

### 2.3 BCAAs catabolism

Branched-chain amino acids (BCAAs) are essential AAs that cannot be synthesized *in vivo* and must be obtained from the diet. The three primary BCAAs are leucine, isoleucine, and valine. BCAA aminotransferase (BCAT1) initiates the catabolic metabolism of BCAAs, facilitating the transfer of amino from BCAAs to other keto acids and generating branched-chain keto acids (BCKAs). Subsequently, BCKA dehydrogenase (BCKD) catalyzes BCKAs. BCKD kinase (BCKDK) phosphorylates and inactivates BCKD, while the mitochondrion-localized protein phosphatase-2C (PP2Cm) specifically dephosphorylates and activates BCKD (Nie et al., 2018). Through a series of reactions, *in vivo* isotopic tracing shows that BCAAs produce Ac-CoA and succinyl-CoA, engaging in the TCA cycle (Neinast et al., 2019).

With regard to the kidney, proteomics revealed a significant decrease in the protein levels of BCAA catabolic enzymes, including BCKD E1 subunit alpha (BCKDHA), BCKD E1 subunit beta (BCKHB), mitochondrial medium-chain specific acyl-CoA dehydrogenase (ACADM), isovaleryl-CoA dehydrogenase (IVD), 3-hydroxyisobutyryl-CoA hydrolase (HIBCH), and methylcrotonoyl-CoA carboxylase (MCCC1, MCCC2), in LPS-induced AKI in mice kidney (Xu et al., 2023). The gene expression of kidney BCAAs catabolism-related genes, such as *Bckdha*, *Bckdhb*, *Acadm*, *Mut*, *Ivd*, *Hibch*, *Mccc1*, and *Mccc2*, significantly decreased in Cisplatin, aristolochic acid I-induced and UUO-induced AKI (Piret et al., 2021). Similarly, the expression of *Bckdha*, *Bckdhb*, and *Ppm1k* genes was downregulated in injured proximal tubules (Li H. et al., 2022). In addition, the gene expression of BCAA catabolic enzymes positively correlated with eGFR in human species (Piret et al., 2021). Further experiments revealed that BCKDHB knock out reduced mitochondrial ATP synthesis, and that the BCAA catabolism enhancer BT2 improved tubular ferroptosis (Sone et al., 2023) and increased the oxygen consumption rate in HK-2 cells (Piret et al., 2021). Activation of BCAA catabolism improved renal fibrosis, epithelial-mesenchymal transition and inflammation in DKD mice (Deng et al., 2025). These findings demonstrated that BCAA catabolism was suppressed in AKI, potentially exacerbating the energy shortage and affecting cell death. In addition, the catabolism of BCAAs also affects macrophages; the BCAT1 inhibitor ERG240 downregulated oxygen consumption and glycolysis in LPS-treated macrophages, leading to a less pro-inflammatory phenotype (Papathanassiu et al., 2017). Inhibition of BCAT1 reduced glomerular crescents, serum creatinine, and proteinuria levels by suppressing macrophage infiltration in crescentic glomerulonephritis rats (Papathanassiu et al., 2017). Additionally, Shen J et al. found that cerebral BCAAs accumulated due to microbiota changes in ischemic stroke rats. BCAAs activated the protein kinase B/activator of transcription/NF-κB axis, exacerbating microglia-induced neuroinflammation (Shen et al., 2023).

Impaired BCAA catabolism leads to the accumulation of BCAAs and BCKAs, The concentration of BCAAs increased in mice kidney cortical tissue during UUO (Li H. et al., 2022), while kidney isoleucine and valine concentrations were elevated in LPS (Xu et al., 2023)- and cardiopulmonary bypass with deep hypothermic circulatory arrest (CPB/DHCA) (Davidson et al., 2022)-induced AKI. In rats with cisplatin-induced AKI, urine leucine and valine levels increased (Pariyani et al., 2017). In human, urinary leucine was proven to be positively correlated with the degree of contrast-induced acute kidney injury (Cheng et al., 2022), and another study showed increased urinary valine and leucine have a good prediction efficacy for pediatric AKI (Muhle-Goll et al., 2020). By contrast, another study reported a decrease in urine and kidney BCAAs during UUO-induced injury (Jouret et al., 2016), as well as a decrease in isoleucine and valine in plasma during UIRI injury (Shan et al., 2023). These discrepancies may arise from variations in disease stages, experimental methodologies, or tissue-specific metabolic responses.BCAAs, particularly leucine, mainly transmit signals by targeting mTOR pathway, participating in protein synthesis, cell proliferation, inflammation, and oxidative stress in various tissues and cell types (Zhenyukh et al., 2017; Yamamoto et al., 2017; Zhang et al., 2016). BCKAs, in a dosage-dependent manner, promoted mitochondrial peroxide production (Sun et al., 2016). Moreover, BCAAs impact pathological outcomes by regulating metabolic pathways. Accumulated BCAAs enhanced FAO by activating the general control nonderepresible-2 (GCN2)/activating transcription factor-6 (ATF6)/peroxisome proliferation-activated receptor alpha (PPAR-α) pathway (Li et al., 2020) and suppressed glucose oxidation by inhibiting pyruvate dehydrogenase activity (Li et al., 2017) in IR-induced myocardial injury. In the liver, BCAAs suppressed lipogenesis by blocking protein kinase B2/sterol regulatory element-binding protein/insulin-induced gene 2a signaling and enhanced glycogenesis by regulating protein kinase B 2/FOXO1 signaling (Zhao et al., 2020). In the kidney, BCAAs supplementation reduced GFR and renal plasma flow, increased plasma free fatty acids, and kidney smooth muscle actin collagen levels in 5/6 nephrectomy rats (Pillai et al., 2019). A L-Leucine-rich diet reduced kidney glomeruli numbers in a doxorubicin toxicity model (Duarte et al., 2023). However, another study demonstrated that the administration of BCAAs downregulated serum creatinine at D28 after UUO in rats, suggesting that BCAAs might partially prevent UUO-induced AKI (Jouret et al., 2016). Nonetheless, it remains unclear whether excessive BCAAs activate signaling transduction or enhance BCAA catabolism to compensate for the energy shortage in AKI.

### 2.4 Tryptophan metabolism

Tryptophan undergoes metabolism through three ways: the kynurenine pathway, the indole pathway, and the serotonin pathway (Hui et al., 2023). In the kynurenine pathway, tryptophan transforms into quinolinic acid which is then catalyzed by the speed-limiting enzyme quinolinate phosphoribosyltransferase, which is abundant in TECs. The process results in the *de novo* synthesis of nicotinamide adenine dinucleotide (NAD), an electron acceptor in TCA cycle. However, Mehr et al. discovered a downregulation of quinolinate phosphoribosyltransferase expression in mice, leading to increased renal and urine quinolinic acid levels and a decrease in NAD levels, and this shift in metabolism contributed to the adverse outcomes of IR-induced AKI (Poyan Mehr et al., 2018). Furthermore, other metabolites of tryptophan, such as kynurenine and indoxyl sulfate, activate the aromatic hydrocarbon receptor, initiating oxidative stress and inflammation that mediate kidney pathogenesis (Hui et al., 2023).

## 3 Upregulated metabolic pathways

### 3.1 Glycolysis

**FIGURE 2:**
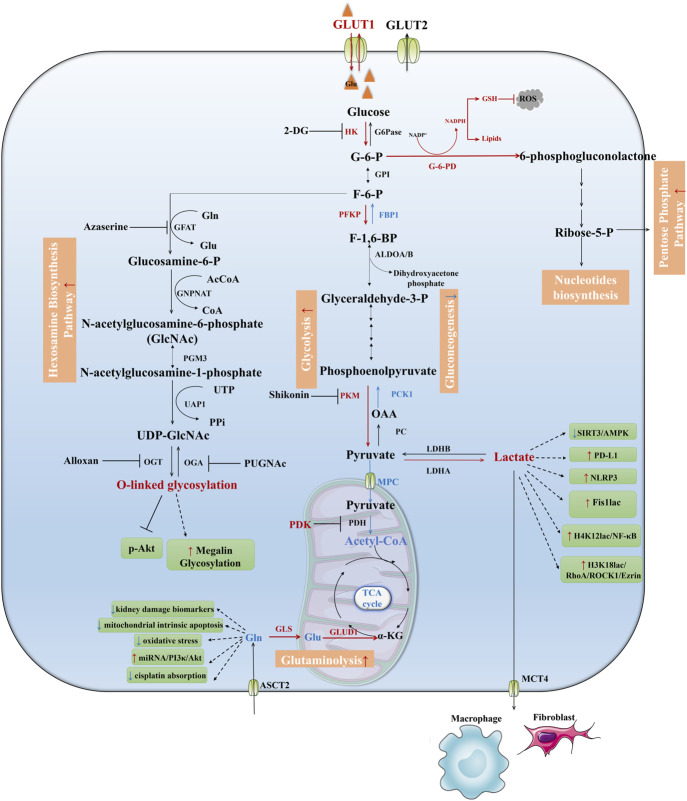
Downregulated pathways in TECs during AKI. Fatty acids are transported into the mitochondria for oxidation as acylcarnitines through the activity of carnitine CPT1 and CPT2, then acyl-CoA undergoes stepwise oxidation to Ac-CoA and enters the TCA cycle which coupling with oxidative phosphorylation to produce ATP. This process is shut down during AKI which leads to maladaptive tubule repair. Ketolysis, BCAA catabolism, NAD *de novo* biosynthesis from tryptophan were suppressed during AKI as well. IDH, Isocitrate dehydrogenase; A-KGDH, α-Ketoglutaric acid dehydrogenase; SCS, Succinyl-CoA synthetase; FH, Fumarase; SDH, Succinic dehydrogenase; MDH, Malate dehydrogenase; CS, Citrate synthase; SCD, Stearoyl-CoA Desaturase.

Glycolysis is a process that catabolizes glucose and supplies ATP without oxygen in the cytosol (Figure 2). Glucose uptake is mainly mediated by sodium glucose transporter 1/2 from the tubular lumen into proximal tubular cells, and glucose transporter type 1/2 which lies on the basolateral membrane and is responsible for transferring glucose intracellularly to the interstitium, or the reverse. Glucose is initially phosphorylated by hexokinase to glucose-6-phosphate (G-6-P). After a series of reactions, G-6-P produces phosphoenolpyruvate, which is then catalyzed by pyruvate kinase M2 to generate pyruvate. Pyruvate is transferred into mitochondria and catalyzed by pyruvate dehydrogenase to produce Ac-CoA, participating in the TCA cycle under normoxia conditions. By contrast, under hypoxic conditions, pyruvate is catalyzed by lactate dehydrogenase to form lactate. The early proximal TECs reabsorb glucose from the tubule lumen, but owning to scarce hexokinase, they hardly use glucose as energy substrate. The situation is reversed in medulla TECs due to low oxygen tension in healthy conditions (Klein et al., 1981; Uchida and Endou, 1988).

A metabolomic shift occurs in TECs during AKI to compensate for the energy shortage resulting from the mitochondrial dysfunction-induced FAO and oxidative phosphorylation deficiency. It was shown that glycolysis is upregulated in various models of AKI, including cecal ligation and puncture-, IRI-, and cisplatin-induced AKI (Lan et al., 2016; Gómez et al., 2017; Xie et al., 2023). Tubular hypoxia-induced factor-1α activation enhanced glucose transporter type 1 mRNA expression, which means hypoxia may facilitate glucose transporter type 1-mediated glucose uptake from the basolateral side for glycolysis (Farsijani et al., 2016). On one hand, IRI kidneys exhibited elevated levels of lactate, increased expression of glycolysis enzymes including hexokinase 2, phosphofructokinases, and pyruvate kinase M2 (Lan et al., 2016; Legouis et al., 2020), increased activity of hexokinase, and decreased expression of gluconeogenesis enzymes fructose-1,6-bisphosphatase 1 and phosphoenolpyruvate carboxykinase (Legouis et al., 2020). Lactate dehydrogenase has been widely used as a biomarker for predicting AKI (Heidari Beigvand et al., 2021; Popov et al., 2017; Bagshaw et al., 2007). 2-Deoxy-D-glucose (2-DG), the inhibitor of hexokinase, has been shown to promote autophagy, suppress apoptosis, and improve kidney function by enhancing SIRT3/AMPK pathway in LPS-induced AKI (Tan et al., 2021). Additionally, the pyruvate kinase M2 inhibitor shikonin rescued kidney fibrosis in UUO-induced injury, reducing tubular apoptosis, fibrosis, and fibroblast and macrophage infiltration, whereas it did not attenuate fibrosis in mouse proximal tubular cell line (Wei et al., 2019). On the other hand, mitochondrial pyruvate channel was downregulated during AKI, indicating a decrease in mitochondrial pyruvate uptake (Rauckhorst et al., 2023). Pyruvate dehydrogenase (PDH), the gateway enzyme linking glycolysis with TCA cycle, is inactivated when phosphorylated by pyruvate dehydrogenase kinase. Lan et al. showed that PDH phosphorylation was upregulated in the IRI mice kidney (Li et al., 2020). Additionally, Oh et al. found that the proximal tubule cell-specific knockout of pyruvate dehydrogenase kinase 4 (PDK4) or pharmacological inhibition of PDK alleviated IR-induced kidney injury by reducing succinate levels in tubules (Zhao et al., 2016). This concurrently mitigated oxidative stress, elevated mitochondrial membrane potential and ATP production, together with reduced mitochondrial fragmentation (Oh et al., 2023; Wei et al., 2019). These findings indicated that pyruvate tends to be directed toward anaerobic lactate production in TECs rather than incorporation into the aerobic TCA cycle. This metabolic shift contributes to worsening kidney function in AKI.

As the final product of glycolysis, lactate levels significantly increased in AKI models, including the CPB/DHCA (Davidson et al., 2022), IRI (Lan et al., 2016), cisplatin (Pariyani et al., 2017), and sepsis (Tan et al., 2021) models. It has been repeatedly shown that lactate serves as a critical risk factor for AKI (Liu Z. et al., 2019; Zhao et al., 2022). Moreover, lactate plays various roles in contributing to the progression of AKI. Tan et al. found that lactate attenuated the expression of SIRT3 and phosphorylated AMPK, inhibited autophagy and enhanced apoptosis in LPS-treated HK-2 cells (Tan et al., 2021). Lactate also upregulated programmed cell death ligand 1 expression, suppressing the immune response in sepsis-induced AKI in mice (Xu et al., 2021). In addition, lactate can participate in post-translational modelling as the donor, with lactylation primarily determined by the concentration of lactate and the activity of glycolysis (Zhang et al., 2019). Lactate-dependent modification occurred both in histone proteins and non-histone proteins and exerting diverse effects during AKI. Renal protein lactylation activated the NLR family pyrin domain containing 3 inflammasome and promoted the transition from AKI to CKD (Yu et al., 2023). Wang et al. found that the key glycolytic enzyme, 6-phosphofructo-2-kinase/fructose-2,6-biphosphatase 3 (PFKFB3) lead to the accumulation of lactate during ischemia-reperfusion injury which would increase histone 4 lysine 12 lactylation which further activate NF-κB signaling pathway and result in kidney fibrosis (Wang et al., 2024). Sheng et al. found that the downregulation of the deacetylase SIRT3, mediated hyperacetylation and inactivation of pyruvate dehydrogenase E1 subunit alpha 1 (PDHEα1), leading to increased lactate levels in TECs. The additional lactate led to elevated lactylation and increased levels of mitochondrial fission 1 protein, lysine 20, which subsequently led to excessive mitochondrial fission and ATP depletion in septic AKI mice (An et al., 2023).

Moreover, excessive lactate can be excreted into the interstitial microenvironment and is absorbed by monocarboxylate transporter, or activates G protein-coupled cell receptor 81 to interact with interstitial cells such as fibroblasts (Jin et al., 2021) and inflammatory cells (Yang et al., 2022), potentially affecting kidney function. Lactate derived from TECs is absorbed by fibroblasts via monocarboxylate transporter 1, promoting fibroblast activation and proliferation in folic-acid-induced AKI (Shen et al., 2020). The administration of lactate significantly reduced the extracellular acidification rate in LPS-treated macrophages. Increased lactylation of pyruvate kinase M2 pyruvate kinase M2 at the K62 site promoted pyruvate kinase M2 tetramerization, and inhibited pyruvate kinase M2 translocation into the nucleus. This suppression of glycolysis results in macrophage polarization toward a reparative phenotype (Wang J. et al., 2022).

To summarize, AKI enhances glycolysis in TECs, acting as an alternative source of energy supply during low oxygen availability. However, continuous glycolysis and the accumulation of lactate aggravates tubular injury, promoting AKI progression.

### 3.2 Pentose pyruvate pathway

The pentose phosphate pathway catabolizes glucose without ATP production. Glucose is catalyzed by G-6-PDH so as to biosynthesize 5-ribose phosphate for nucleotide production and generate nicotinamide adenine dinucleotide phosphate (NADPH) from the electron acceptor NADP^+^. Scantlebery et al. observed a significant upregulation of pentose phosphate pathway-related genes, including glucose‐6‐phosphate 1‐dehydrogenase, transaldolase 1, transketolase, and ribose‐phosphate diphosphokinase 1, following IR-AKI in mice kidneys. The upregulated NADPH levels may serve as a reductive agent in fatty acid and cholesterol synthesis, leading to lipotoxicity in TECs (Scantlebery et al., 2021). On the other hand, a *Nature* study revealed that inhibition of the glycolysis enzyme pyruvate kinase M2 compels glucose flux toward the pentose phosphate pathway in proximal tubular cells (Zhou et al., 2019), and upregulated NADPH could mitigate damage caused by reactive oxygen species, protecting against IR-induced kidney injury (Zhou et al., 2019). Moreover, G-6-PDH-deficient mice exhibited higher proteinuria, oxidative stress, and lower NADPH and glutathione levels in the kidney (Xu et al., 2010). These apparently dual functions of the pentose phosphate pathway necessitate further research to fully elucidate its role in AKI.

### 3.3 Hexosamine biosynthetic pathway

The hexosamine biosynthetic pathway is another branched process of glucose metabolism. Firstly, glucose is metabolized to G-6-P by hexokinase, then phosphohexose isomerase catalyzes G-6-P to fructose-6-phosphate. Glutamine fructose-6-phosphate amidotransferase (GFAT) deaminizes glutamine and produces glucosamine-6-phosphate, then acetyl-CoA and Uridine-5′-triphosphate (UTP) are introduced into this process to generate uridine 5′-diphospho-N-acetyl-D-glucosamine. Finally, O-linked N-acetylglucosaminyltransferase (OGT) and O-GlcNAcase serve as “writer” and “reader” to add or remove O-linked β-N-acetylglucosamine moieties (O-GlcNAc) on serine or threonine residues of proteins. This process is known as O-GlcNAcylation which also post-translationally modifies protein activity and function.

The hexosamine biosynthetic pathway and its metabolites are augmented and play cytoprotective roles during acute stress periods (Zachara et al., 2004). Hu et al. confirmed that enhanced O-GlcNAc signaling transduction reduced oxidative stress and cell apoptosis through inhibiting phosphorylation of protein kinase B. Inhibition of this process using alloxan, an OGT inhibitor, worsened kidney function in contrast-induced-AKI (Hu et al., 2017). Another study showed that remote ischemia preconditioning attenuated oxidative stress and tubular apoptosis through enhancing the hexosamine biosynthetic pathway and O-GlcNAc glycosylation levels in contrast-induced AKI, while pharmacological inhibition of GFAT abolished this protective effect (Hu et al., 2018). However, long-term augmentation of O-GlcNAcylation tended to harm kidney function. Spontaneously hypertensive rats exhibited that hyper-O-GlcNAcylation, GFAT and OGT level were positively correlated with proteinuria, while inhibiting GFAT reduced proteinuria. This could be attributed to reduced protein reabsorption in tubular cells due to O-GlcNAcylated megalin (Silva-Aguiar et al., 2018).

### 3.4 Glutaminolysis

Glutaminolysis is the catabolic process of glutamine. Glutamine is catalyzed by glutaminase, resulting in the generation of glutamate. Subsequently, glutamate is deaminated by glutamate dehydrogenase to produce α-ketoglutarate. The catabolism of glutamine also serves as a component of anaplerosis, that aims to maintain the homeostasis of intermediates within the TCA cycle. Glutaminolysis functions as an alternative pathway, providing the carbon skeleton for biosynthesis and energy expenditure during nutrition stress.

Metallo et al. found that MRC-5 cells (human lung fibroblasts) treated with isotope-labeled glutamine were heavily reliant on reductive glutamine metabolism to synthesize Ac-CoA for lipid synthesis in hypoxia environments (Metallo et al., 2011). In cardiac studies, researchers observed a significant increase in glutaminase-1, the speed-limiting enzyme in glutaminolysis, during angiogenesis II-induced hypertrophy and proliferation in cardiomyocytes and fibroblasts. Correspondingly, the inhibition of glutaminase-1 prevented pathological cardiac remodeling by suppressing anaplerosis from glutamine, thereby impeding the biosynthesis of nucleic acids and lipids in mice (Yoshikawa et al., 2022). However, other studies also showed that glutaminase-regulated glutaminolysis also elevated the NADPH/NADP^+^ ratio as well as glutathione levels against oxidative stress, which is beneficial for cell growth and proliferation (Tong et al., 2021; Yang et al., 2017).

Emerging evidence suggests metabolic rewiring involving enhanced glutaminolysis may occur during AKI progression. Single-cell RNA sequencing revealed an increase in the expression of the glutaminolysis enzyme *Gls* and *Glud1* genes in proximal tubules in both uIRI and UUO mice (Li H. et al., 2022; Kirita et al., 2020), this transcriptional signature indicating enhanced glutaminolysis in kidney TECs (single-cell sequencing database: http://humphreyslab.com/SingleCell/). Nowik et al. demonstrated that the uptake and breakdown of glutamine increased to maintain an acid-base balance in acidosis-induced AKI (Nowik et al., 2008). Aligned with metabolomic profiling, Wei et al. found concentrations of glutamine decreased in the renal cortex and plasma in IRI- (Wei et al., 2014) and LPS-induced AKI (Xu et al., 2023), suggesting accelerated glutaminolytic flux in AKI. However, contradictory results have been reported in other studies, where some showed an increase in serum glutamine levels in cecal ligation and puncture-induced AKI in a pig model (Izquierdo-Garcia et al., 2019) and in LPS-induced AKI (Gao et al., 2021).

Glutaminolysis may also impact inflammatory cells; T cells exhibited metabolic reprogramming during IRI, with upregulated glutaminase activity during the ischemia period and a decline during reperfusion. Applying a glutaminase antagonist mitigated both IRI- and cisplatin-induced kidney function deterioration by inhibiting the activation and proliferation of T cells (Lee et al., 2023). Nevertheless, there is insufficient evidence to assert that glutaminolysis undergoes changes in TECs and affects the progression of AKI, making it an area worth exploring. Collectively, enhanced glutaminolysis appears detrimental to myocardial cells in pathological conditions, and renal glutaminolysis gene expression is upregulated in uIRI and UUO mice kidneys. However, the precise role of glutaminolysis in TECs and AKI remains unclear.

Numerous studies have demonstrated that the administration of glutamine can offer protection against AKI. In a randomized controlled trial, glutamine supplementation was found to significantly reduce kidney damage biomarkers in cardiac surgery patients at a high risk of AKI (Weiss et al., 2023). Mechanistically, glutamine attenuated IRI-induced AKI by modulating the glutamine gamma glutamyltransferase 2/heat shock protein 70/apoptosis signal-regulating kinase/c-Jun N-terminal kinase pathway and diminished mitochondrial intrinsic apoptosis in TECs (Thomas et al., 2022). Glutamine reduced oxidative stress in gentamicin-induced AKI in rats (Zhan et al., 2022), targeted microRNA/Notch and microRNA/phosphoinositide-3-kinase/protein kinase B signaling pathways in IR rats (Li S. et al., 2022), reduced organic cation transporter2 expression, and reduced the absorption of cisplatin HK-2 cells (Kim et al., 2015), among other mechanisms.

### 3.5 Polyamine metabolism in AKI

Polyamines, which include putrescine, spermidine, and spermine, are essential for cell proliferation, chromatin organization, gene regulation, cell death and immune system functions (Holbert et al., 2022). The synthesis of polyamine is initiated by the speed-limiting enzyme ornithine decarboxylase, which converts L-ornithine to putrescine. Subsequently, the addition of an aminopropyl group is carried out by spermidine synthase and spermine synthase, leading to the production of spermidine and spermine. The catabolism of polyamine involves the spermidine/spermine N1-acetyltransferase/N1-acetylpolyamine oxidase cascade. Additionally, spermine can be directly oxidized to spermidine by spermine oxidase, while amine oxidase copper-containing 1 (AOC1) is responsible for the breakdown of putrescine. Polyamines and their metabolites entering the circulation can be utilized by cells throughout the body, thereby affecting microenvironments.

Sieckmann et al. found that the key synthetic enzyme ODC1 was downregulated, while the catabolic enzyme AOC1 was upregulated in multiple kidney injury models, including IRI, UUO, kidney transplantation, rhabdomyolysis, and streptozocin-induced diabetes (Sieckmann et al., 2023). The increased AOC1 was secreted into the bloodstream, where it further catabolized putrescine into toxins. Interestingly, the knockout of AOC1 didn’t affect kidney function but led to a reduction in cortical tubules with casts and downregulation of the injury marker lipocalin-2 in IRI mice (Sieckmann et al., 2023). Another study demonstrated an elevation in the expression of the speed-limiting enzyme in polyamine and the activity of spermine/spermidine N1-acetyltransferase and spermine oxidase, two critical enzymes in polyamine catabolism, in the kidneys during IR (Zahedi et al., 2014), septic (Xu et al., 2023), and cisplatin- (Zahedi et al., 2017) induced AKI. Inhibiting either of these enzymes resulted in DNA injury, inflammatory reaction (Zahedi et al., 2014), oxidative stress (Wang et al., 2004), and endoplasmic reticulum stress/unfolded protein response (Zahedi et al., 2017). In summary, polyamine catabolism was heightened in AKI, and blocking polyamine catabolism alleviated kidney injury.

Accordingly, due to the enhanced catabolism of polyamine, the concentrations of putrescine increased, and spermidine and spermine decreased in tetracycline treated HEK-293 cells (Wang et al., 2004). Kidney putrescine levels increased in LPS (Xu et al., 2023)- and CPB/DHCA (Davidson et al., 2022)-induced AKI, while putrescine, spermidine, and spermine all increased in both kidney and serum (Gao et al., 2021; Sieckmann et al., 2023). Notably, urine spermidine showed a high concentration in cardiac surgery-associated AKI and displayed a strong association with the AKI outcome (Martin-Lorenzo et al., 2021).

## 4 Potential therapeutic target of metabolic pathways in treating AKI

Several studies have investigated drugs targeting these metabolic pathways to thwart the progression of AKI (Table 2).

**TABLE 2:** Potential therapeutic targets of metabolic pathways in AKI.

Drug name	Drug species	Mechanisms of action	Corresponding evidence
AICAR and metformin	AMPK activator	Optimizing cellular ability to coordinate OXPHOS and glycolysis	Sepsis-induced AKI in mice (Jin et al., 2020)
Sanglifehrin A	Cyclophilin binding compound	Inhibiting interaction between cyclophilin D and PPARα, increasing FAO	Cisplatin-induced AKI in mice (Jang et al., 2020)
Harmine	Twist1 inhibitor	Downregulating FAO enzymes including CPT1a and activating PGC1α	IRI- and UUO-induced AKI in mice (Liu et al., 2022)
αKlotho	FGF23 receptor	Suppressing ubiquitin-mediated degradation of adipose triglyceride lipase, enhancing adipose triglyceride lipase-mediated lipolysis and lipophagy	IRI-induced AKI in mice (Wang et al., 2023)
Dapagliflozin	SGLT2 inhibitor	Suppressing hypoxia-induced factor-1α and preventing the metabolic shift from FAO to glycolysis	STZ-induced tubular injury in mice (Cai et al., 2020)
SGLT2 inhibitor	SGLT2 inhibitor	Decreasing the rate of transepithelial glucose uptake, glycolysis and gluconeogenesis	Diabetes-induced proximal tubule injury in human (Schaub et al., 2023; Tuttle, 2023)
Anarodustat	HIF-1 activator	Enhancing glycolysis and glycogenesis, NADPH and glutathione	IRI-induced AKI in rats (Schaub et al., 2023; Tuttle, 2023)
Shikonin	PKM2 inhibitor	Suppressing glycolytic enzyme PKM2	UUO-induced AKI in mice (Schaub et al., 2023; Tuttle, 2023)
2-DG	HK inhibitor	Inhibiting glycolysis and enhancing SIRT3/AMPK pathway	LPS-induced AKI in mice (Schaub et al., 2023; Tuttle, 2023)
GSK2837808	LDH inhibitor	Reducing lactate production	Sepsis-induced AKI in mice (Schaub et al., 2023; Tuttle, 2023)
Oxamate	LDH inhibitor	Inhibiting glycolysis and production of lactate in TECs and suppressing fibroblast activation	Folic acid-induced AKI in mice (Schaub et al., 2023; Tuttle, 2023)
NAM	NAD precursor	Augmenting production of the fat breakdown product β-hydroxybutyrate, leading to increased production of prostaglandin PGE2	IRI-induced AKI in mice (Schaub et al., 2023; Tuttle, 2023)
L. casei Zhang	Probiotic	Altering SCFAs and nicotinamide metabolism	IRI-induced AKI in mice (Schaub et al., 2023; Tuttle, 2023)
HA-KTP/PSPD/siRNA	Renal-targeted gene delivery system	Silencing Arg-2	Drug-induced AKI in mice (Schaub et al., 2023; Tuttle, 2023)

AICAR, 5-Aminoimidazole-4-carboxamide1-β-D-ribofuranoside; AMPK, AMP-activated protein kinase; AKI, acute kidney injury; Arg-2, Arginase 2; HA-KTP, kidney targeting peptide-modified hyaluronic acid; HK, hexokinase; IRI, ischemia reperfusion injury; NAD, nicotinamide adenine dinucleotide; NAM, nicotinamide; LPS, lipopolysaccharide; OXPHOS, oxidative phosphorylation; PKM2, pyruvate kinase M2; SCFA, short chain fatty acid; SIRT3, sirtuin 3; STZ, streptozocin; PSPD; UUO, unilateral ureteral obstruction.

Hypoxia-induced factor 1 (HIF-1) enhances glycolysis by increasing the expression of all glycolytic enzymes, including hexokinase 1, hexokinase 2, 6-phosphofructokinase, liver type, phosphofructokinase, platelet, aldolases (ALDA and ALDC), glyceraldehyde 3-phosphate dehydrogenase, and pyruvate kinase. Additionally, hypoxia-induced factor 1b suppresses FAO, reduces oxidative phosphorylation, and mitochondrial oxygen consumption. It also increases PDK1 and reduces cellular acetyl-CoA levels (Taylor and Scholz, 2022). In diabetic kidney disease, proximal tubules also exhibit metabolic shift from FAO to glycolysis which is associated with increased hypoxia-induced factor-1α. By suppressing HIF-1α by the sodium-glucose cotransporter-2 inhibitor (SGLT2i), dapagliflozin, prevented the metabolic shift from FAO to glycolysis in TECs (Cai et al., 2020). Indeed, applying the HIF-1 activator anarodustat, which inhibits the prolyl hydroxylase domain, rescued low-oxygen-treated HK-2 cells, though anarodustat enhanced glycolysis, which also increased glycogenesis, NADPH and glutathione, preserving sufficient glucose for energy supply and providing substrates to counteract oxidative stress (Ito et al., 2020). This underscores the importance of considering metabolic disorder as a complex, systemic, and interactive process when implementing interventions.

Employing single-cell RNA sequencing to explore differentially-expressed genes in kidney tissue from young patients with Type 2 diabetes and controls, Schaub et al. found that SGLT2i decreased the rate of transepithelial glucose uptake. This resulted in decreased glycolysis and gluconeogenesis in the proximal tubule, suppressing the activation of mTOR complex 1 and mitigating diabetes-induced proximal tubule injury (Schaub et al., 2023; Tuttle, 2023).

Liu L et al. found that Twist1, a transcription factor implicated in fibrotic pathogenesis across multiple organs, was upregulated in uIR- and UUO-induced kidney injury. Conditional knockout of twist1 in proximal tubular cells reversed the downregulation of FAO enzymes, including acyl-coenzyme A oxidase 1 and CPT1a, by activating peroxisome proliferator-activated receptor-γ coactivator 1-α (PGC1-α), thereby alleviating fibrosis in AKI mice (Liu et al., 2022). Furthermore, Harmine, a Twist1 inhibitor, prevented fatty acid metabolic disorders and fibrogenesis, suggesting Twist1 could be a potential therapy target for AKI in the future.

αKlotho suppressed ubiquitin-mediated degradation of adipose triglyceride lipase, enhancing adipose triglyceride lipase-mediated lipolysis and lipophagy, subsequently protecting mice from IRI-induced AKI (Wang et al., 2023).

Probiotics were also been investigated as metabolic modulator in acute kidney injury. For instance, probiotic *Lactobacillus* casei Zhang (L. casei Zhang) could alter SCFAs and nicotinamide metabolism and protect kidney injury in IRI-induced AKI (Zhu et al., 2021).

Newly developed technology regulating the expression of metabolic enzyme was also applied in AKI. Utilizing spermine (SPD) as a monomer for siRNA delivery to downregulate Arg-2 expression, and assembling with kidney targeting peptide (KTP)-modified hyaluronic acid (HA) to improve the *in vivo* delivery and renal targeting of the gene vector. This system showed the alleviation of kidney injury through the mechanisms including promotion of mitochondrial autophagy, mitigation of oxidative stress, and inhibition of apoptosis in drug-induced AKI (Gu et al., 2024).

Numerous other interventions have attempted to target metabolic upstream molecules such as AMPK (Jin et al., 2020; Harley et al., 2022), peroxisome proliferator-activated receptor gamma, peroxisome proliferator-activated receptor-γ coactivator 1α (Tran et al., 2016), and others. Additionally, other interventions have focused on post-translational modifications of metabolites, including histone deacetylases (Guo et al., 2019), and SIRT (Huang et al., 2022).

## 5 Conclusion and perspectives

**FIGURE 3:**
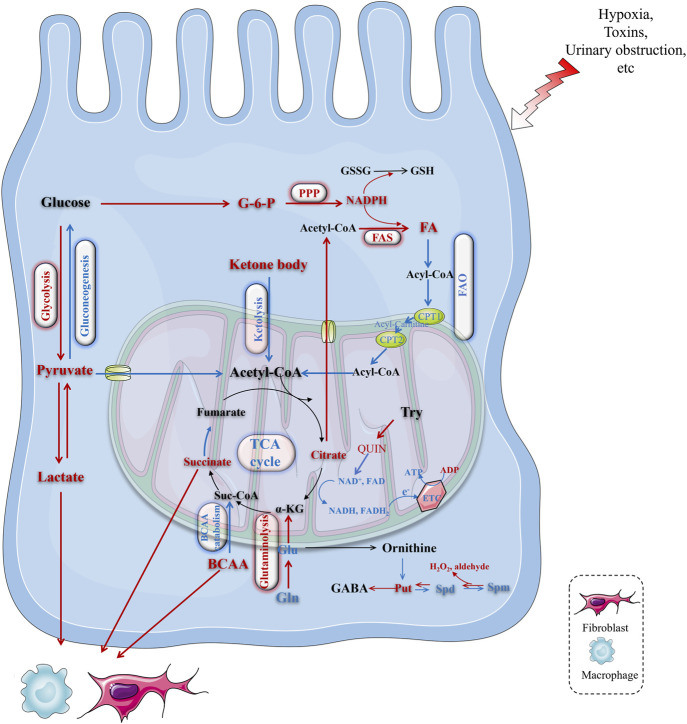
Upregulated pathways in TECs during AKI. Glycolysis, pentose phosphate pathway, hexokinase biosynthesis pathway, glutaminolysis pathway were enhanced during AKI. And their final products or intermediate metabolites played different roles during AKI. HK, hexokinase; G-6-P, glucose‐6‐phosphate; F-6-P, fructose‐6‐phosphate; GFAT, glutamine fructose-6-phosphate amidotransferase; phosphoenolpyruvate (PEP), then PEP is catalyzed by pyruvate kinase M2 (PKM2); fructose-1,6-bisphosphatase 1 (FBP1), phosphoenolpyruvate carboxykinase (PCK1/2), PC, pyruvate carboxylase.

Accumulating evidence has recently unraveled that the metabolic pathway and energy substrate were switched during AKI. Catabolism of fatty acids, ketone bodies and BCAAs was shut down and replaced with enhanced glycolysis, glutaminolysis, pentose pyruvate pathway, and polyamine catabolism (Figure 3; Table 1). The reprogrammed metabolic pathway aims to compensate for energy shortages during the early stages of AKI, however, it tends to be detrimental for TEC repair. The differential metabolites triggered by metabolic rewriting also affect the kidney outcome mechanistically.

In addition, metabolic heterogeneity across AKI etiologies still need to be verified, some evidence indeed suggests distinct energy utilization patterns among IRI, sepsis, nephrotoxin-induced AKI, driven by their unique pathophysiological mechanisms. IRI was characterized by abrupt ATP depletion due to hypoxia, proximal tubules shift to anaerobic glycolysis (increased PFK-1 and lactate accumulation). Mitochondrial dysfunction persists even post-reperfusion, with impaired fatty acid β-oxidation (CPT1 downregulation) and ROS-induced damage to ETC complexes. Sepsis-induced AKI was mainly mitochondrial dysfunction and systemic inflammatory-driven metabolic reprogramming. Despite preserved renal blood flow, microcirculatory shunting creates “cytopathic hypoxia.” Proinflammatory cytokines (e.g., TNF-α, IL-6) induce Warburg-like metabolic reprogramming–enhanced glycolysis (HIF-1α stabilization) but suppressed OXPHOS. Mitochondrial uncoupling (UCP2 upregulation) further reduces ATP yield. Lipolysis and amino acid catabolism are amplified to fuel gluconeogenesis. During nephrotoxin-induced AKI, ROS-mediated disruption of OXPHOS and substrate-specific toxicity. Direct tubular toxicity disrupts mitochondrial integrity (reduced cristae density, cytochrome c release). For instance, Cisplatin inhibits Complex I/IV and Krebs cycle enzymes (aconitase suppression), blocking both glucose and fatty acid metabolism. Persistent NADPH oxidase activation exacerbates oxidative stress, impairing redox-sensitive metabolic sensors (AMPK/PGC-1α axis). Despite distinct initiating insults, different etiologies of AKI ultimately converge on overlapping pathophysiological mechanisms in TECs, resulting in strikingly similar metabolic reprogramming during advanced injury stages.

Given that metabolic pathways are dynamic, reversible, systemic, and intricate processes, researchers commonly employ a combination of proteomics, metabolomics, and transcriptomics to analyze enzyme expression, metabolite concentration, and isotope-labeled metabolites for tracking outlets. Recently, single-cell RNA sequencing and spatial metabolomics have provided new insights into the transcriptome and metabolism of various cell types. However, even with the integration of different methodologies, accurate and consistent answers for metabolic pathways may not always be achieved. Therefore, additional studies are imperative to further elucidate the role of metabolic pathways that may underly disease processes in the kidney.

In conclusion, numerous studies have demonstrated that metabolic rewriting is inevitable in AKI. This review delved into the dual roles of adaptive metabolic shifts and their maladaptive consequences during AKI, aiming to inspire researchers to prioritize metabolic insights into clinically diagnostic/prognostic biomarkers and precision therapies.
